# Hypothalamic Leptin Receptor‐Expressing Neurons: A Regulator of Anxiety‐Feeding Imbalance in Animal Models of Anorexia Nervosa

**DOI:** 10.1002/mco2.70632

**Published:** 2026-02-11

**Authors:** Jingjia Liang, Yanrong Zheng, Zhong Chen

**Affiliations:** ^1^ Zhejiang Key Laboratory of Neuropsychopharmacology, School of Pharmaceutical Sciences Zhejiang Chinese Medical University Hangzhou China

1

In a recent study published in Nature Neuroscience, Figge‐Schlensok et al. [1] identified a novel role for lateral hypothalamic leptin receptor‐expressing (LepR^LH^) neurons in balancing anxiety and feeding. Their findings might explain why individuals with anorexia nervosa (AN) prioritize weight control over physiological hunger: their LepR^LH^ neurons may fail to appropriately balance anxiety and feeding motivation.

AN is a psychiatric disorder with the highest mortality rate and characterized by restrictive eating, body image disturbance, and substantial weight loss. Approximately 90% of cases occur in young females. Numerous clinical trials have tested various pharmacological interventions and cognitive‐behavioral therapy for AN; however, these approaches have limited efficacy. Moreover, patients frequently experience high relapse rates (approximately 50%) 12 months after discharge. Comorbid anxiety is recognized as a major driver of the disorder. The presence of any anxiety disorder, rather than specific anxiety subtypes, increases vulnerability to eating pathology. Evidence suggests that leptin contributes to anxiety‐feeding interactions. Polymorphisms in the leptin receptor gene directly correlate with susceptibility to AN. Administration of recombinant human leptin to patients with AN results in symptomatic improvement, including reduced weight‐related anxiety during hospitalization [2]. Moreover, the lateral hypothalamic (LH) area may represent a critical brain region for regulating the anxiety‐feeding balance. In addition to regulating metabolic demands during hunger states, LH has been identified as a region that responds to maladaptive anxiety and can modulate chronic‐pain‐related behavioral responses through feeding [3]. Therefore, clarifying the activity regulation of LepR^LH^ neurons during anxiety states and their connectivity with anxiety‐related brain regions may disclose novel therapeutic targets for AN intervention.

Nevertheless, the regulation of feeding behavior by LepR^LH^ neurons is state‐dependent. Activation of these neurons exerts minimal impact on food intake under satiated conditions, whereas knockout of *LepR* in LH increases food consumption. Conversely, activating the LepR^LH^ neurons during hunger reduces food intake. This state‐dependent regulatory mechanism raises critical safety concerns: is the role of LepR^LH^ neuronal activation in AN affected by hunger status and could modulation of these neurons under different physiological states potentially transform therapeutic benefits into adverse outcomes? Further research is required to address these issues.

Figge‐Schlensok et al. [1] also identified projections from the prefrontal cortex (PFC) to the LH as critical regulators of the function of LepR^LH^ neurons, with this modulation particularly pronounced in high‐anxiety states. The PFC, a key region for executive function and anxiety processing, contains neurons that selectively inhibit LepR^LH^ neuronal activity in high‐anxiety mice. Improved LepR^LH^ neuronal activity functionally counteracts PFC inputs, enabling feeding in anxiogenic environments. Although severe anxiety typically impairs feeding, mice can employ adaptive strategies to reduce food‐related anxiety. Furthermore, stimulus‐evoked LepR^LH^ responses in the activity‐based anorexia (ABA) model exhibited strong anxiety‐ and state‐dependent differences: compared with the habituation phase, running‐wheel (RW)‐evoked LepR^LH^ activity increased dynamically across successive RW visits during the restriction phase (summarized in Figure 1). Nevertheless, under high‐anxiety conditions, ABA mice demonstrated slower development of RW‐induced excitation, resulting in overall lower activation levels than those under low‐anxiety conditions. Consequently, anxiety‐related PFC‐mediated over‐inhibition of LepR^LH^ neuronal cells in AN prolongs the time required for these neurons to reach sufficient activation levels through running to exert anxiolytic effects. This delay may result in excessive exercise, thereby increasing the anxiety levels and further impairing the feeding behavior, which provides a neurobiological explanation for the anxiety‐driven feeding inhibition observed in AN.

**FIGURE 1:**
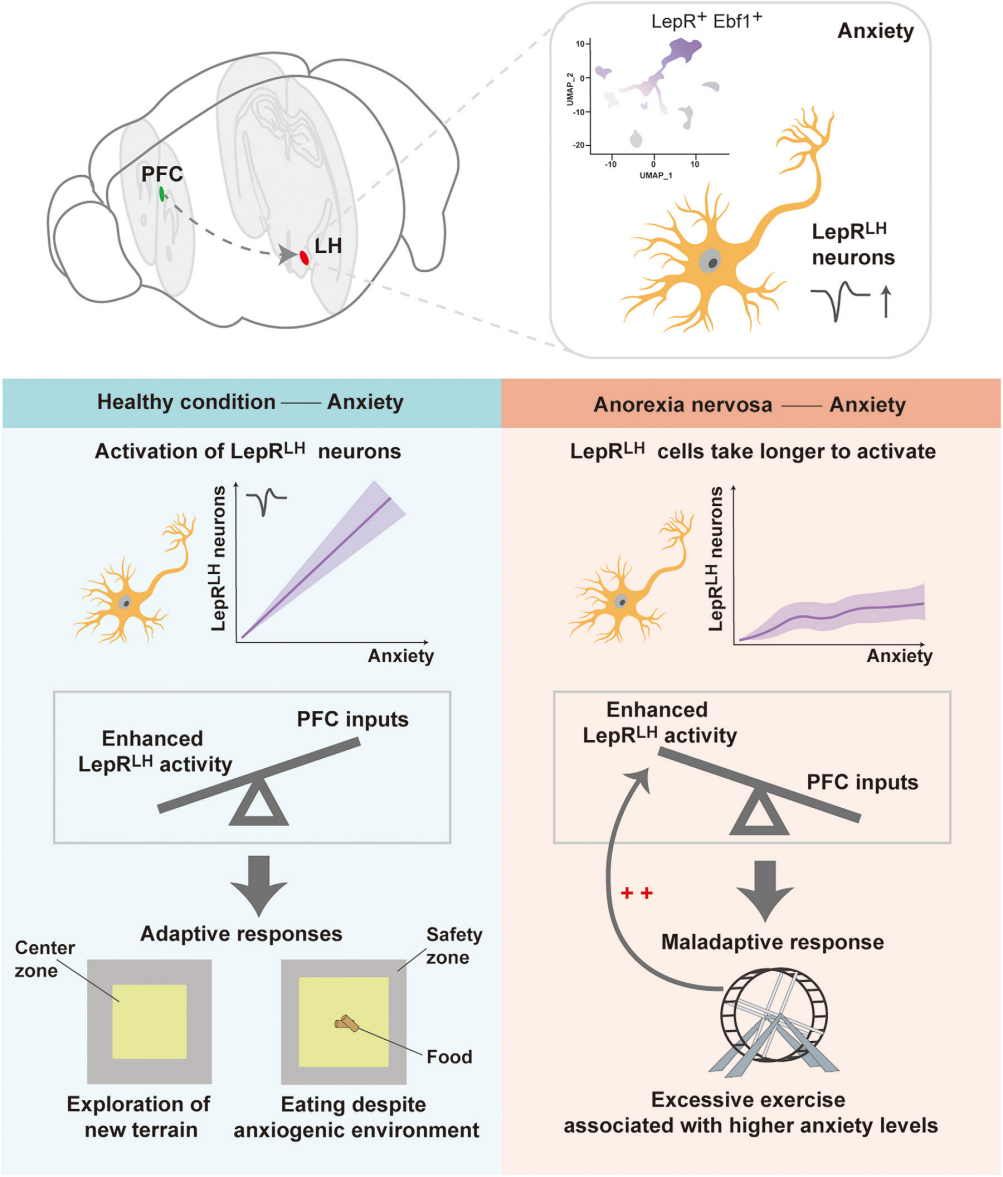
LepR^LH^ encode anxiogenic stimuli to enable adaptive behavioral responses despite anxiogenic conditions both in health conditions and in anorexia nervosa model. Under health conditions, LepR^LH^ neurons can be activated by anxiety‐inducing stimuli, promoting adaptive behavior; during the restrictive phase in anorexia model animals, LepR^LH^ neurons require longer activation times during running to achieve the same level of activation as in health conditions. This delay may contribute to excessive exercise and heightened anxiety levels. LepR^LH^, lateral hypothalamic leptin‐receptor‐expressing neurons; PFC, prefrontal cortex; Ebf1, early B‐cell factor 1.

A recent study also reported that food restriction significantly reduces the expression of brain‐derived neurotrophic factor (BDNF) in the PFC [4]. This alteration cannot be reversed even through gradual refeeding, despite the functional recovery of other brain regions such as the dorsal striatum. BDNF is crucial for synaptic plasticity and cognitive flexibility, and its deficiency in the PFC may correlate with the impaired decision‐making and abnormal threat information processing observed in patients with AN via neuroimaging [5]. During food restriction, the expression of TrkB—the high‐affinity receptor for BDNF—is upregulated in the PFC [4], which may sensitize the PFC‐LH circuit to anxiety signals by amplifying residual BDNF signaling or inducing receptor‐mediated hypersensitivity. Consequently, a pathological cycle emerges: BDNF deficiency and TrkB upregulation in the PFC may synergistically improve the inhibitory tone of the PFC‐LH pathway, thereby suppressing the function of LepR^LH^ neurons and exacerbating anxiety‐feeding dysregulation. This hyperactive PFC circuit, potentially driven by irrational fears of weight gain or catastrophic thoughts concerning food, may pathologically suppress the inherent anti‐anxiety drive of LepR^LH^ neurons and forces the inhibition of the brain's intrinsic capacity to overcome anxiety for the purpose of eating.

Although the study identified LepR^LH^ neurons as a central hub that counteracts anxiety to support adaptive feeding responses, critical gaps remain in understanding AN pathogenesis and developing effective treatments. First, through transcriptomic analysis of LH single‐cell RNA‐sequencing data, the authors revealed molecular heterogeneity within the LepR^+^ population and identified a LepR^+^ cluster (cluster 3) with increased expression of early B‐cell factor 1 (Ebf1), which is associated with anxiety disorders and AN. However, whether these neurons play distinct roles in anxiety‐feeding regulation and how LepR proteins influence neuronal activity remain to be investigated, as current conclusions are primarily based on observed neuronal activity changes. Second, the specific neurotransmitters released by LepR^LH^ neurons that mediate anxiety reduction and promote feeding remain unknown. Characterizing these signaling molecules would establish a foundation for developing targeted pharmacological interventions. Finally, additional clinical studies are necessary to investigate the correlation between the therapeutic effects of leptin in AN and functional magnetic resonance imaging (fMRI)‐measured activation patterns within the homologous human LH circuit.

In conclusion, the investigation by Figge‐Schlensok et al. provides novel insights on the potential role of LepR^LH^ neurons in anxiety‐feeding regulatory networks, enhancing our understanding of the pathophysiology of AN. Moreover, this study shifts the therapeutic focus from targeting feeding switches to modulating the anxiogenic brake that regulates the anxiety‐homeostatic interaction, thereby providing a new direction for developing treatments that directly address the core anxiety pathology of the disorder.

## Author Contributions

Jingjia Liang wrote the manuscript and prepared the figure. Yanrong Zheng and Zhong Chen discussed and revised the manuscript. All authors have read and approved the final manuscript.

## Ethics Statement

The authors have nothing to report.

## Conflicts of Interest

The authors declare no conflict of interest.

## Data Availability

The authors have nothing to report.
